# Susceptibility to auditory hallucinations is associated with spontaneous but not directed modulation of top-down expectations for speech

**DOI:** 10.1093/nc/niac002

**Published:** 2022-02-01

**Authors:** Ben Alderson-Day, Jamie Moffatt, César F Lima, Saloni Krishnan, Charles Fernyhough, Sophie K Scott, Sophie Denton, Ivy Yi Ting Leong, Alena D Oncel, Yu-Lin Wu, Zehra Gurbuz, Samuel Evans

**Affiliations:** Department of Psychology, Durham University, Durham, UK; Department of Psychology, Durham University, Durham, UK; Department of Psychology, University of Sussex, Brighton, UK; Centro de Investigação e Intervenção Social, Instituto Universitário de Lisboa (ISCTE-IUL), Lisbon, Portugal; Department of Psychology, Royal Holloway, University of London, London, UK; Department of Psychology, Durham University, Durham, UK; Institute of Cognitive Neuroscience, University College London, London, UK; Department of Psychology, Durham University, Durham, UK; Department of Psychology, Durham University, Durham, UK; Department of Psychology, Durham University, Durham, UK; Department of Psychology, Durham University, Durham, UK; Department of Psychology, Durham University, Durham, UK; Department of Psychology, University of Westminster, London, UK

**Keywords:** predictive coding, psychosis, speech perception, speech-in-noise, audition

## Abstract

Auditory verbal hallucinations (AVHs)—or hearing voices—occur in clinical and non-clinical populations, but their mechanisms remain unclear. Predictive processing models of psychosis have proposed that hallucinations arise from an over-weighting of prior expectations in perception. It is unknown, however, whether this reflects (i) a sensitivity to explicit modulation of prior knowledge or (ii) a pre-existing tendency to spontaneously use such knowledge in ambiguous contexts. Four experiments were conducted to examine this question in healthy participants listening to ambiguous speech stimuli. In experiments 1a (*n* = 60) and 1b (*n* = 60), participants discriminated intelligible and unintelligible sine-wave speech before and after exposure to the original language templates (i.e. a modulation of expectation). No relationship was observed between top-down modulation and two common measures of hallucination-proneness. Experiment 2 (*n* = 99) confirmed this pattern with a different stimulus—sine-vocoded speech (SVS)—that was designed to minimize ceiling effects in discrimination and more closely model previous top-down effects reported in psychosis. In Experiment 3 (*n* = 134), participants were exposed to SVS without prior knowledge that it contained speech (i.e. naïve listening). AVH-proneness significantly predicted both pre-exposure identification of speech and successful recall for words hidden in SVS, indicating that participants could actually decode the hidden signal spontaneously. Altogether, these findings support a pre-existing tendency to spontaneously draw upon prior knowledge in healthy people prone to AVH, rather than a sensitivity to temporary modulations of expectation. We propose a model of clinical and non-clinical hallucinations, across auditory and visual modalities, with testable predictions for future research.

## Introduction

Hallucinations have long been considered a product of top-down processes: what the mind brings to our perception of the world, not the other way round (Esquirol 1832). Auditory verbal hallucinations (AVHs) in particular have been studied extensively because of their association with schizophrenia, occurring in 60–90% of cases (Bauer *et al.* 2011) and at rates that are often double those seen for other modalities (Waters *et al.* 2014). AVHs have been proposed to result from various internal sources such as memories, imagery, and self-talk or inner speech (Mintz and Alpert 1972; Waters *et al.* 2003; Seal *et al.* 2004). Difficulties in distinguishing the internal from external were interpreted as a problem with ‘reality monitoring’, in which disruptions to source monitoring could explain how self-generated cognitive states could become perceptual experiences (Feinberg 1978; Bentall 1990; Frith 1992). Although not always framed as a ‘top-down’ model of hallucinatory experience, this grounded much research in the metacognitive domain, consistent with cognitive approaches to psychosis in clinical practice (Morrison *et al.* 1995).

Recent interest in predictive processing approaches has reframed the putative role of top-down processes in hallucination. Under the predictive processing framework (PPF), all of perception and cognition is the result of a trade-off between generative models of the world, shaped by prior expectations and prediction error, i.e. the gap between expectation and sensory input (Clark 2013; Hohwy 2014). Hallucinations have been posited as an imbalance between prior expectation and prediction error (Fletcher and Frith 2009; Jardri and Denève 2013; Powers *et al.* 2016). Such accounts have been argued to be consistent with source-monitoring theories (Wilkinson 2014; Griffin and Fletcher 2017; Corlett *et al.* 2019) and may even reflect a generalization of prediction mechanisms inherent in earlier theories (Pickering and Clark 2014). Nevertheless, they involve a shift in emphasis away from the metacognitive monitoring of self, focusing instead on expectation and learning as being central to hallucination.

Supporting evidence for a PPF approach to hallucinations was provided by Teufel *et al.* (2015), in a study of individuals with an at-risk mental state for psychosis. Patients and healthy controls were asked to discriminate monochrome Mooney (1957) images, before and after exposure to their original templates (pictures of humans and animals). While both groups improved their discrimination after viewing the templates, clinical participants showed significantly enhanced discrimination compared to controls, consistent with top-down information being given greater weight in their perceptual processing. Teufel and colleagues then replicated this finding in a sample of 40 healthy participants rated for psychosis-proneness on measures of hallucination-like experiences (*r* = 0.42, the Cardiff Anomalous Perceptions Scale; Bell *et al.* 2006) and delusional traits (*r* = 0.33, the Peters Delusion Inventory; Peters *et al.* 2004), with higher scores on these scales being associated with a greater improvement in discrimination following exposure to the templates (Teufel *et al.* 2015).

These findings speak to visual processes—but what of voices, the most common kind of hallucination in psychosis? Various source-monitoring studies have demonstrated biases in auditory signal detection in people with hallucinations—often on white noise tasks (Bentall and Slade 1985a)—but facilitatory effects like those described by Teufel and colleagues have not typically been studied.

Analogous to Mooney images, sine-wave speech (SWS; Remez *et al.* 1981; Rosen *et al.* 2011) is a perceptually ambiguous stimulus derived from speech that allows for exploration of top-down effects on perception. SWS is not usually identified as intelligible speech by naïve listeners; instead, it requires prior training to be recognized and understood. In a recent study, a sample of non-clinical voice-hearers (NCVH)—individuals with frequent AVH but no need for clinical care (Johns *et al.* 2014; Peters *et al.* 2016)—were scanned in fMRI while naïvely listening to SWS (Alderson-Day and Lima *et al.* 2017). Instead of being told to listen for speech, participants were instructed to listen for an unintelligible target sound amidst a range of SWS stimuli. Despite this, a majority of the NCVH group identified speech in the SWS spontaneously and without any training. When asked to estimate the point at which they recognized the hidden speech (visual markers had been displayed indicating numbered ‘rounds’ during the scan), NCVH participants reported doing so significantly earlier than a matched control group. Subsequent tests of discrimination following the ‘reveal’ that speech was present, failed to identify any group differences. This suggested that voice-hearers may automatically draw upon top-down resources—such as speech templates—when faced with ambiguous sensory input (Alderson-Day *et al.* 2017).

Both experiments are consistent with top-down processing being linked to hallucinations, but they highlight contrasting effects: a *modulatory* effect (Teufel *et al.* 2015) and a *naïve listening* effect on perception (Alderson-Day *et al.* 2017). They also differ in design and stimuli, making it challenging to directly compare them. In this paper, we aimed to draw together these effects, adapting the SWS procedure across a series of experiments with healthy participants to explore top-down effects on audition. We began with the original SWS stimuli used in Alderson-Day *et al.* (2017) deployed in two parallel experiments examining modulation effects (Experiments 1a and 1b). In Experiment 1a (*n* = 60), we followed a similar test-train-test procedure to Teufel and colleagues, using the CAPS (Bell *et al.* 2006) and PDI (Peters *et al.* 2004) to measure unusual perceptual experiences and delusional beliefs. Experiment 1b (*n* = 60), run in parallel, used an alternative measure specific to AVH-proneness: a version of the Launay–Slade Hallucination Scale-Revised (Bentall and Slade 1985b; Morrison *et al.* 2000). It also included participants intentionally recruited to expand the potential range of individual differences in hallucination-proneness (specifically, people with a history of imaginary companions; Fernyhough *et al.* 2019), and an added condition that sought to further prime potential templates for speech. Based on the modulation hypothesis, we hypothesized that improvements in discrimination following template exposure should be associated with higher hallucination-proneness.

One problem with SWS is that some participants attempting to understand it may go from not understanding it all before training to suddenly understanding it all, while others may spontaneously learn to decode it. This learning profile can make it hard to compare with perceptual learning for Mooney images. So, in Experiment 2 (*n* = 99), we tested the same modulation effect but with a new stimulus, sine-vocoded speech (SVS). We developed this particular stimulus set with the aim of offering a tighter control on some of the potential learning effects inherent to SWS comprehension—making it more comparable to Teufel *et al.* (2015). As in experiments 1a and 1b, we expected that modulation of discrimination would be related to hallucination-proneness.

Finally, having tested modulation effects using SVS, we returned to the behavioural design from Alderson-Day *et al.* (2017), examining SVS perception under naïve listening conditions (Experiment 3, *n* = 134). According to the naïve listening hypothesis, we predicted that hallucination-proneness would be higher in those who were quicker to recognize that SVS contained hidden speech. After the naïve listening procedure, we also tested them on their memory for the hidden words, therefore providing a more objective test of spontaneous decoding of the hidden speech. Data and analysis code for each of the experiments are available via OSF.

## Experiment 1a

Modulating prior knowledge of sine-wave speech.

The aim of our first experiment was to develop a modulation of expectation in the auditory modality and to see how this related to hallucination-proneness scores. In contrast to Teufel *et al.* (2015), who used 12 blocks of before/after trials, we chose to play all 90 trials, train on the whole set, and then retest for all trials (a ‘one-shot’ procedure). This was chosen to minimize any potential training effects occurring across multiple blocks of testing and training. We predicted that higher CAPS scores would be associated with greater increases in discrimination following template exposure. We also explored this effect for delusion-proneness scores on the PDI.

## Method

### Participants

A convenience sample of 60 participants was recruited from a university cohort (age *M*(*SD*) = 21.22 (3.11), range 18–32 years, 18 male).^1^ Individuals were invited to take part if they were native English speakers with no hearing impairments or any previous psychiatric or neurological diagnoses. Participants received course credit or a gift voucher in recognition of their time. For this and the remaining experiments, written informed consent was obtained for all participants and all procedures were approved by a university ethics committee.

### Materials and procedure

#### SWS discrimination task

**Figure 1.**
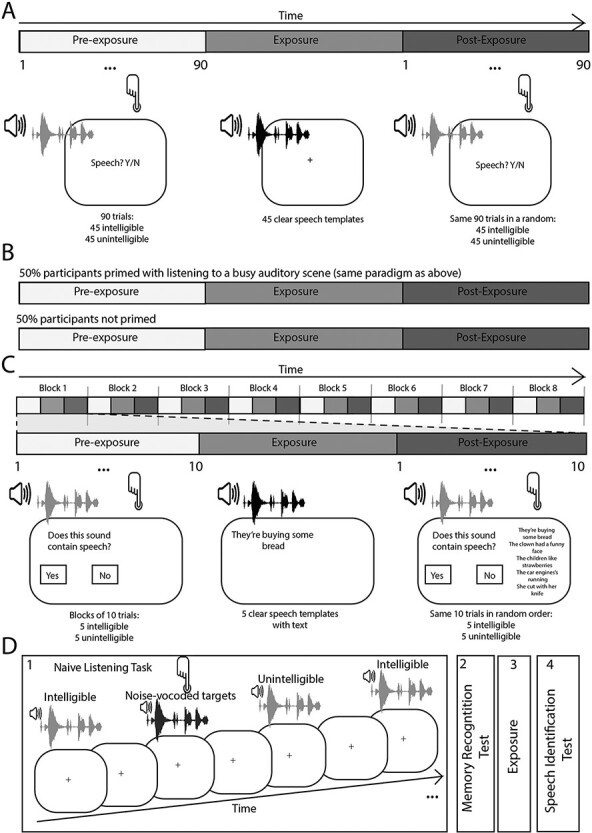
Overview of experiments. (A) Experiment 1a: Participants heard 90 trials comprised of potentially intelligible and unintelligible sounds and judged whether each sound contained speech (pre-exposure). They were then exposed to the target clear speech exemplars from which the intelligible trials were made (exposure) and then asked again to judge which trials contained speech (post-exposure). (B) Experiment 1b: Participants took part in the same paradigm as Experiment 1a but half the participants were primed by listening to a busy auditory scene and the other half were not. (C) Experiment 2: Participants heard blocks of 10 trials using the same pre-exposure, exposure, post-exposure cycles in Study 1. (D) Experiment 3: Participants took part in a naïve listening experiment in which they were tasked with identifying sounds with a specific acoustic quality (noise-vocoded sounds). They were not informed that some sounds contained speech. They were then asked whether they had heard any speech in the naïve listening task and took part in a memory recognition test to see if they remembered the intelligible trials. They were then exposed to the clear speech targets and tested on their identification of speech

SWS is created by tracking and modeling the formant tracks of spoken sentences using a sine-wave tone. This procedure can be used to create potentially intelligible stimuli (in which the frequency and amplitude tracks of the same original sentence are combined)—or unintelligible stimuli (combining the frequency and amplitude information of two different sentences). Both are typically perceived as unintelligible, but potentially intelligible SWS typically becomes comprehensible following training and exposure to 2–3 template sentences (Rosen *et al.* 2011). Experiment 1 used the same SWS stimuli as in Alderson-Day *et al.* (2017), which were first developed by Rosen *et al.* (2011).^2^ The original sentences were taken from the Bamford-Kowal-Bench (BKB) sentence set (Bench *et al.* 1979). Participants completed the task in a quiet university room. The task was presented using Psychtoolbox in MATLAB 2016 on a Windows PC with a 17" monitor, using Sennheiser headphones for stimulus delivery. See Fig. 1 for a summary of the design of this and the other reported experiments.

The SWS discrimination task was divided into two runs of 90 trials (45 intelligible SWS, 45 unintelligible SWS) occurring *before* and *after* participants heard each of the original sentences that the intelligible SWS trials were based on (‘template exposure’). On each run, participants listened to 2.5s clips of SWS and were asked to decide whether the speech was present or not for each trial, allowing for signal detection measures to be calculated based on hit rates (intelligible trials marked as containing speech) and false alarm rates (unintelligible trials marked as containing speech). Signal detection theory (Stanislaw and Todorov 1999) was used to calculate discrimination (*d′*), plus two measures of bias: criterion (*C*) and beta (*β*), the measure most typically used in source monitoring research on hallucinations (Brookwell *et al.* 2013). Where hit rates and false alarms were 0 and 1, the Macmillan and Kaplan (1985) method was used (i.e. zero scores replaced with 0.5/*n* and 1 replaced with (*n*−0.5)/*n*).

#### Questionnaires

In each experiment, questionnaires were collected after task-based measures were taken.

The *Cardiff Anomalous Perceptions Scale* (CAPS; Bell *et al.* 2006) is a commonly used scale of hallucination-proneness that assesses a range of unusual perceptual experiences—including auditory, visual and gustatory phenomena—across 32 items. It correlates with other measures of schizotypy and hallucinations, such as the Oxford Liverpool Inventory of Feelings and Experiences (OLIFE; Mason *et al.* 1995), and has strong internal reliability (alpha = 0.87). Participants are asked to indicate whether they have ever had a specific experience, and if so, how distressing, how intrusive, and how frequent the experience was (on a 1–5 scale). To assess the general tendency to experience hallucinations, here we used the total frequency as the main CAPS outcome.

The *Peters Delusion Inventory—21 item version* (PDI; Peters *et al.* 2004) is a shortened adaptation of the original 40-item PDI (Peters *et al.* 1999). Both measures have been used extensively as a measure of proneness to unusual beliefs in the general population, have good convergent validity with other measures of schizotypy, and have strong internal reliability (e.g. alpha > 0.8). The PDI has an identical structure to the CAPS (the latter being modelled on the former). Frequency of belief was included as the main outcome.

Our analytic approach sought to first assess changes in discrimination and bias variables following the exposure phase, using paired *t*-tests. We then (i) followed Teufel and colleagues’ analysis by testing the relationship between CAPS scores and d-prime improvement using correlational analysis, and (ii) used partial correlation to test this association while controlling for confounds such as baseline performance. Correlations with other change scores (i.e. beta and *C*), relations to pre-exposure performance and associations with the PDI were included for exploratory purposes.

## Results and discussion

Table 1 shows signal detection outcomes for the SWS discrimination task. As would be expected, performance significantly improved following exposure to the original (i.e. non-masked) sentences, as indicated by an increase in *d′*. However, bias also significantly increased, with participants being more likely to say that speech was present after template exposure [before hits *M*(*SD*)% = 67.9% (21.1%), false alarms *M*(*SD*) = 20.6% (12.3%); after hits *M*(*SD*)% = 88.6% (14.2%), false alarms = 26% (14.9%)].

**Table 1.** Signal detection outcomes for the SWS discrimination task in experiment 1a

	Before		After				
	*M*	*SD*	*M*	*SD*	Change statistic *T(Z)*	*P*	*d(r)*
*d′*	1.47	0.77	2.17	0.81	−8.63	4.809e−12	−0.90
*C*	0.19	0.45	−0.37	0.44	−6.25	4.212e−10	0.81
*β*	1.63	1.87	0.60	0.51	−5.74	9.202e−09	0.74

Note: Higher values of *d*ʹ indicate increased sensitivity to detect speech. Scores below 0 for *C* and 1 for beta indicate a bias to indicate speech is present. Wilcoxon tests were used for *C* and beta due to non-parametric data.

### Testing a modulation effect via bivariate correlation

**Figure 2.**
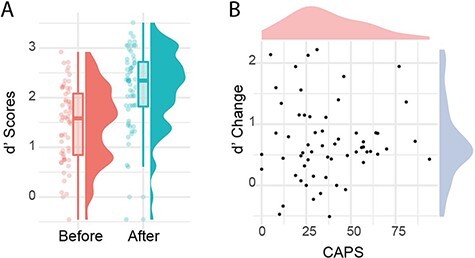
Comparing discrimination pre- and post-template exposure (A) and the relation of performance change to hallucination-proneness (B)

Following Teufel *et al.* (2015), we first tested for bivariate correlations between change in *d′*, CAPS, and PDI scores. Despite the clear change in discrimination following exposure, no correlation was observed between CAPS scores and change in *d′* (Pearson’s product, *r* (58) = 0.02, *P *= 0.864, 95% CI = −0.23:0.28), contrary to the modulation hypothesis (see Fig. 2). A one-sided Bayesian analysis (using JASP v.0.8.6 with default priors) indicated a BF of 0.19 for the experimental hypothesis and 5.39 for the null (i.e. good evidence for a lack of any effect of interest). A similar result was evident for PDI scores (*r* (58) = 0.11, *P* = 0.400).

### Controlling for baseline performance and other confounds

Analysis of difference scores can be affected by the values at baseline (Senn 2006) and are often thought to be less reliable than the measures they derive from. To account for this, we also ran partial correlation tests controlling for baseline discrimination scores, using the ppcor package in R (Kim 2015). This adjustment made no difference to the results (*r* = −0.04, *P* = 0.758) suggesting that the overall null result was unlikely to be driven by baseline performance differences.

### Relations to bias

Finally, we also tested for any relations between bias (*C* and *β*) and proneness to psychotic experiences. No significant correlations were observed between task and questionnaire scores (all *P* > 0.10; see Supplementary Materials).

The results of Experiment 1a, therefore, did not support the idea of a modulatory effect of expectation being related to hallucination-proneness. When exposed to new information via the original sentence templates, participants consistently performed better in terms of their speech vs. non-speech discrimination and increased their bias to state that speech was present (across intelligible and unintelligible SWS stimuli). None of these performance changes were related to hallucination-proneness scores on the CAPS or delusion ratings on the PDI.

Two limitations are important to consider. The first is that using hallucination-proneness in non-clinical analogue samples has been questioned for its ability to identify individuals with truly hallucinatory experiences (Stanghellini *et al.* 2012). If valid, this could lead to the concern that correlations between tasks and self-report will be very low and very hard to capture, given the low base level and minimal variation in proneness scores. The spread of CAPS scores shown in Fig. 2 is comparable to prior research of this kind and not insubstantial when compared to clinical data (Bell *et al.* 2006). Nevertheless, directed recruitment of members of the general population with higher levels of hallucination-proneness could provide greater variation and more opportunity to examine how changes in expectation relate to unusual sensory experiences.

A second concern is that measures of hallucination-proneness can yield inconsistent results, and there is currently no ‘gold standard’ for assessing such experiences in the general population. For the purposes of replication, we used the CAPS but could have instead included the Launay–Slade Hallucination Scale (Bentall and Slade 1985b) which is arguably a more commonly used scale in prior research on hallucinations. Moreover, the CAPS asks about hallucinations across a range of modalities, whereas prevalence rates for AVH—and the auditory nature of the SWS task—may warrant a more specific measure of auditory hallucination-proneness.

## Experiment 1b

Modulating prior knowledge with a wider range of hallucination-proneness.

Experiment 1b, run in parallel to the first, was designed to address potential concerns about the level and specificity of hallucination-proneness. First, as an alternative to the CAPS, we used a revised version of the Launay–Slade Hallucination Scale (Morrison *et al.* 2000), with a specific focus on auditory experiences (McCarthy-Jones and Fernyhough 2011). Second, intending to gather a wider range of unusual experiences, we explicitly set out to recruit individuals with a history of having imaginary companions (ICs). Engaging with imaginary companions has been proposed to bear commonalities with hallucinatory experiences (Pearson *et al.* 2001), even though there is no good evidence that they are a developmental marker for later psychopathology (Taylor 1999; Maijer *et al.* 2019). Specifically, there is evidence to suggest that having an IC as a child is associated with both elevated hallucination-proneness and bias in auditory signal detection skills as an adult (Fernyhough *et al.* 2019). In addition, children with ICs are more likely to hear words amidst jumbled speech, which is similar in many ways to effects seen for SWS (Fernyhough *et al.* 2007).

Finally, we also attempted to provide a second test of the modulation of expectation, by priming half the participants with a short listening activity (listening to a recording of a conversation in a busy room) before attempting the same task as Experiment 1a, i.e. discrimination before and after exposure to the sentence templates. Reasoning that directing participants to listen for speech under suboptimal conditions should prime both the expectation of speech and top-down templates for speech, we predicted that primed participants would go on to show greater speech discrimination of SWS in the subsequent task, even before template exposure. If this could be demonstrated, it would represent a more naturalistic modulation of expectation, by indirectly priming generic speech templates that could assist in the disambiguation of the SWS stimuli. The design for Experiment 1b, therefore, mixed a between-groups approach (prime vs. no-prime) and a within-subjects approach (before vs. after template exposure). As in Experiment 1b, we hypothesized that greater improvements in discrimination scores on the SWS task would be associated with greater LSHS scores.

## Method

### Participants

Sixty participants (age *M*(*SD*) = 23.22 (4.76), range 18–43 years, 14 male) were recruited from university settings, social media, and via word-of-mouth. Exclusion criteria were identical to Experiment 1a. Within the 60, it was possible to recruit 22 people with a history of having imaginary companions as children, of whom 14 were able to provide parental verification of their childhood IC—a validation step considered good practice in IC research (Fernyhough *et al.* 2007, 2019).

### Materials and procedure

The same procedure and task structure were used for the SWS discrimination task as in Experiment 1a. In addition, half of the participants completed a priming activity before the discrimination task. The CAPS and PDI were replaced with a version of the Revised Launay–Slade Hallucination Scale.

#### Listening prime task

Thirty participants were asked to complete the priming activity before the SWS discrimination task. Participants were given a worksheet and were asked to circle words that they heard being mentioned in a 3-minute pre-recorded conversation between five girls. The recording was layered with white noise to increase the difficulty in discerning what was being said. The remaining 30 participants were instructed to close their eyes and count their breaths for 3 minutes in silence, as timed by the experimenter.

#### Questionnaires

Experiment 1b included a version of the *Revised Launay*–*Slade Hallucination Scale* (Morrison *et al.* 2000; McCarthy-Jones and Fernyhough 2011). Since the development of the original scale by Bentall and Slade (1985b), numerous versions of the LSHS have been used to assess hallucination-proneness in the general population. Here we used a five-item version in which all of the items related specifically to auditory experiences, which participants rated for frequency on a scale from 1 (Never) to 4 (Almost Always). This version was developed by McCarthy-Jones and Fernyhough (2011) following a revision by Morrison *et al.* (2000). This version has satisfactory internal reliability (typically alpha => 0.7) and has been used to explore task-to-questionnaire relations in various studies previously (e.g. Garrison *et al.* 2017; Alderson-Day *et al.* 2019).

## Results and discussion

### Testing the priming effect

**Figure 3.**
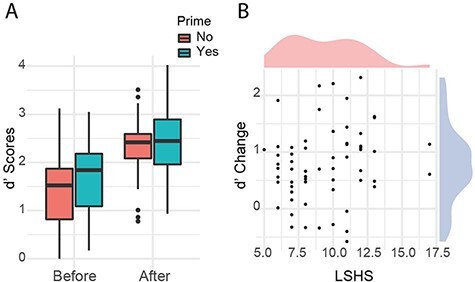
Change in discrimination pre- and post-exposure divided by priming group (A) and relation to hallucination-proneness (B)

A 2 × 2 mixed ANOVA (prime group × pre-/post-exposure) was used to assess the effect of the priming condition on discrimination, plus any interaction it had with exposure to the sentence templates (i.e. before vs. after). As in Experiment 1a, there was a clear increase in discrimination following exposure to the template sentences (*F* (1,58) = 81.31, *P* = 1.24e-12, η^2^*_p_* = 0.58). But despite the manipulation, no main effect of priming was observed on the discrimination task following the listening activity (*F* (1,58) = 0.98, *P* = 0.327, η^2^*_p_* = 0.02, nor any interaction effect between priming group and template exposure (*F* (1,58) = 0.53, *P* = 0.471, η^2^*_p_* = 0.009; see Fig. 3A).^3^ Pre-exposure *d′* scores were very similar in each group [Primed *M*(*SD*) = 1.68 (0.75); Control *M*(*SD*) = 1.45 (0.77)], as were post-exposure scores [Primed *M*(*SD*) = 2.39 (0.79); Control *M*(*SD*) = 2.29 (0.66)]. Comparisons of pre-exposure bias (both *β* and *C*) were also non-significant (all *P* > 0.300; see Supplementary Materials, Experiment 2).

### Testing the modulation effect

Given the lack of differences on any task measure, we subsequently combined the priming groups to facilitate comparison with Experiment 1a. Table 2 shows the SWS task outcomes. As before, there was a significant increase in discrimination following exposure. Pairwise *t*-tests showed that this was also the case for both measures of bias (i.e. lower scores, indicating a greater tendency to say speech is present).

**Table 2.** Signal detection outcomes for the SWS discrimination task in experiment 1b

	Before		After				
	*M*	*SD*	*M*	*SD*	*Change statistic T(Z)*	*P*	*d(r)*
*d′*	1.56	0.76	2.34	0.72	−9.05	9.298e-13	−1.05
*C*	0.29	0.63	−0.42	0.52	9.65	9.59e-14	1.22
*β*	2.58	3.04	0.88	1.62	−5.79	6.741e-09	0.75

Note: higher values of *d′* indicate increased sensitivity to detect speech. Scores below 0 for *C* and 1 for beta indicate a bias to indicate speech is present. Wilcoxon tests were used for beta due to non-parametric data.

The overall mean LSHS-A score of 9.40 places this sample as having a mean level of hallucination-proneness comparable to other samples with childhood histories of ICs, and slightly higher than large samples without any IC history (M = 8.76; Fernyhough *et al.* 2019). As would be expected, participants with an IC in the present sample had significantly higher LSHS-A scores (IC *M*(*SD*) = 10.55 (2.89); No IC *M*(*SD*) = 8.74 (2.41); Z = −2.56, *P* = 0.011, *r* = 0.33).

However, few relations between SWS task outcomes and questionnaire scores were observed even with higher rates of hallucination-proneness in the sample. A stronger relationship was evident between the improvement in *d′* and LSHS-A scores (*r* = 0.22, see Fig. 3B), but this was still non-significant on a Spearman’s test (*P* = 0.089) and Bayesian analysis little evidence for the hypothesized effect (one-sided BF_10_ = 0.81, or 1.25 in favour of the null). Partial correlation, controlling for baseline *d′* scores, also showed no clear association between hallucination-proneness and improvement in performance (*r* = 0.07, *P*= 0.611).

### Relations to bias

Correlations with bias metrics (*C* and *β*) were non-significant (all *r* < 0.21, all *P* > 0.08). Pre-exposure discrimination actually *negatively* correlated with LSHS scores (*r* = −0.34, *P* = 0.008, uncorrected), suggesting more hallucination-prone people were worse at discriminating speech than controls before hearing the sentence templates.

The results of Experiment 1b, therefore, provided a further test of the modulation hypothesis in the auditory domain, but could not support it: improved discrimination following template exposure did not significantly relate to auditory hallucination-proneness even when including individuals with an IC—therefore sampling a broader continuum of hallucination susceptibility. In addition, priming expectation for speech with a further manipulation yielded no difference in discrimination performance. Taken together, the results of experiments 1a and 1b failed to show an effect in the auditory domain comparable to Teufel *et al.*’s modulation effect.

Our findings may suggest that a sensitivity to directed modulation of expectation for speech is not part of the putative mechanism underlying hallucinations. If correct, this would raise problems for a ‘strong priors’ account of the phenomenon (Corlett *et al.* 2019). However, the findings may still reflect unusual properties of SWS itself. For example, SWS comprehension following exposure and training can show something akin to a ‘pop-out’ effect, where suddenly new stimuli can be easily understood. In addition, they offer potentially different opportunities for perceptual learning, compared to Mooney images (Mooney 1957). For the latter, the level of visual noise is high compared to the level of repeated signal across trials, meaning that template exposure is required per image. For SWS, template exposure typically permits more generalized improvements in discrimination across trials (due to similarities in the underlying structure of speech sounds). It is therefore important to tightly control for pre-exposure levels of performance and ensure that difficulty levels are maintained for speech discrimination. In the following experiment, we introduced a new stimulus that could be used to address these issues.

## Experiment 2

Modulating prior knowledge of sine-vocoded speech (SVA) with varying levels of difficulty.

Adapting and extending the SWS developed by Rosen *et al.* (2011) is challenging, as the stimuli were originally hand-edited to closely map the formant contours of speech. To address the factors described above, we deployed a different auditory stimulus in Experiment 2—SVS—and mixed it with unintelligible SVS to add a source of auditory noise. This provided: (i) a way to automate the generation of a degraded speech stimulus (rather than using hand-crafted SWS), allowing the use of a larger number of spoken sentences, and (ii) greater control over manipulating task difficulty. We tested pre- and post-exposure identification of degraded speech in a larger sample and its association with hallucination-proneness at an increased level of difficulty compared to the previous experiments. The task was administered in prior knowledge exposure cycles of ten trials, rather than using a one-shot exposure approach. This was done in part to attempt greater parity with the kind of procedure used by Teufel and colleagues, and to provide greater access to top-down information after exposure within each cycle.

## Method

### Participants

A sample of 99 participants was recruited from university settings, social media, and via word-of-mouth (age *M*(*SD*) = 21.58 (3.34), range 18–34 years, 45 male). Exclusion criteria were identical to the previous experiments. All participants provided informed consent in accordance with the approval of the relevant ethics committee. Due to experimenter error, the questionnaire data were not complete for 12 participants: one participant did not have PDI data and 11 did not have CAPS data. Participants received course credit for taking part.

### Materials and procedure

#### Auditory stimuli

SVS is similar to SWS. However, rather than tracking only the first three formants of speech, the sinewaves are synthesized at the centre frequency of a logarithmically spaced bank of filters spanning a broad frequency range (up to 5 kHz). Like SWS, SVS sentences can be rendered intelligible and recognizable as speech when participants are aware that it is a speech stimulus (Souza and Rosen 2009). SVS can also be rendered unintelligible by flipping the frequency mapping of the original sentence (e.g. pushing energy in high-frequency bands into low bands and vice versa), providing an ideal control stimulus, with similar complexity and acoustic structure.

The BKB sentences (Bench *et al.* 1979) were recorded by a male speaker at a sample rate of 22.05 kHz. Each sentence was digitally filtered using either 8 or 16 bands, with sixth-order Butterworth IIR filters in MATLAB. Filter spacing was based on equal basilar membrane distance (Greenwood 1990) across a frequency range of 100–5000 Hz. Next, the output of each band was half-wave rectified and low-pass filtered (fourth-order Butterworth) at 30 Hz to extract the amplitude envelope. The envelope was then multiplied by a tone carrier at the band centre frequency for each filter. The resulting signal (envelope × carrier) was filtered using the same bandpass filter as for the first filtering stage. RMS level was adjusted at the output of the filter to match the original analysis, and the signal was summed across bands.

When larger numbers of filter bands (e.g. 16 vs. 8) are used to synthesize a spoken sentence this increases the spectral information in the signal with a resulting increase in intelligibility (Souza and Rosen 2009).

Sine-vocoding was used to make two types of stimulus: an intelligible and unintelligible SVS condition. For the Intelligible SVS condition, intelligible SVS sentences were mixed with an unintelligible sine-vocoded sentence that acted as a competing noise source. This was designed to make stimulus identification more difficult reducing learning in the pre-exposure phase and ensuring greater dynamic range in the prior knowledge advantage provided by hearing clear speech templates. The RMS level of the intelligible sentence was rendered at different levels of intensity relative to the unintelligible sentence before mixing them [e.g. a differing signal-to-noise ratio (SNR) was used]. This, alongside manipulating the number of vocoding bands, provided a way to manipulate the difficulty of speech identification.

For the Unintelligible SVS condition, two sentences were sine-vocoded and frequency flipped and mixed in an equivalent SNR as the Intelligible SVS condition, with one unintelligible sentence arbitrarily assigned to be of greater intensity than the other. This ensured that the Intelligible SVS and Unintelligible SVS conditions were of equivalent complexity and overall intensity. A set of stimuli were synthesized from +6 dB to −6 dB in 3 dB steps using 8 and 16 bands. The sentences composing the intelligible and unintelligible conditions were mutually exclusive. We have made this full set of stimulus materials available here (even those conditions not used in this article) to facilitate future research: https://osf.io/yrn9j/.

### Auditory task

Participants attended to sounds presented using MATLAB on a laptop using Sennheiser HD 206 headphones. They were randomly assigned to five groups which each received two different sets of auditory stimuli (approximately 20 participants in each group). These two sets differed in either SNR and/or the number of bands. Informal piloting indicated that the 8 band +6 dB condition provided an appropriately challenging listening level, such that accuracy would be above chance, but not at ceiling. Each group was tested on this common 8 band +6 dB condition, plus one other condition. This common condition was included to pool the data across groups to test for the relationship between signal detection measures and questionnaire responses. The second condition had a different signal-to-noise ratio and/or a different number of vocoding bands and was included to scope how speech detection accuracy was influenced by the SNR and number of bands.

Data from these conditions indicated that the SNR level and the number of bands had a significant effect on participant performance and confirmed the observation that the 8 band +6 dB condition provided an appropriately difficult listening experience (see Supplementary Materials, Experiment 2). The specific set of sentences used in each condition and order of the conditions was counterbalanced across participants to ensure that participants did not hear repetitions of any sentences across the experimental session and to reduce order effects.

Testing in each auditory condition took around 20 minutes (40 minutes total). Before each condition participants received a short training session in which they were introduced to the intelligible and unintelligible stimuli. They were informed that they would hear a 50:50 ratio of intelligible to unintelligible trials in the forthcoming experiment and needed to judge whether each trial contained speech or not.

In the experiment, sounds were presented in blocks of ten in a randomized order (five intelligible trials and five unintelligible trials). In the pre-exposure phase, on each trial participants indicated whether the sentence contained speech or not. They then received exposure to prior knowledge: they heard each target sentence presented in the original clear speech and saw the written transcript of the sentence. In the post-exposure phase, they heard the intelligible and unintelligible sentences that they heard in the pre-exposure phase in a different randomized order and were again asked to indicate which sentences contained speech. The five transcribed target sentences remained on the right-hand side of the screen during the post-exposure phase to reduce memory demands and to maximize the prior knowledge benefit. This cycle repeated 8 times, each time with a different set of 10 sentences, such that 80 trials were presented in each condition (40 intelligible; 40 unintelligible) and 160 trials were presented in total across the experiment.

Following administration of the auditory tasks, total frequency scores were collected for the CAPS (Bell *et al.* 2006) and the PDI (Peters *et al.* 2004). Signal detection measures were calculated as in the previous experiments. The total testing time was around 50 minutes.

## Results and discussion

### Assessing SVS discrimination across trials and at different levels of difficulty

**Figure 4.**
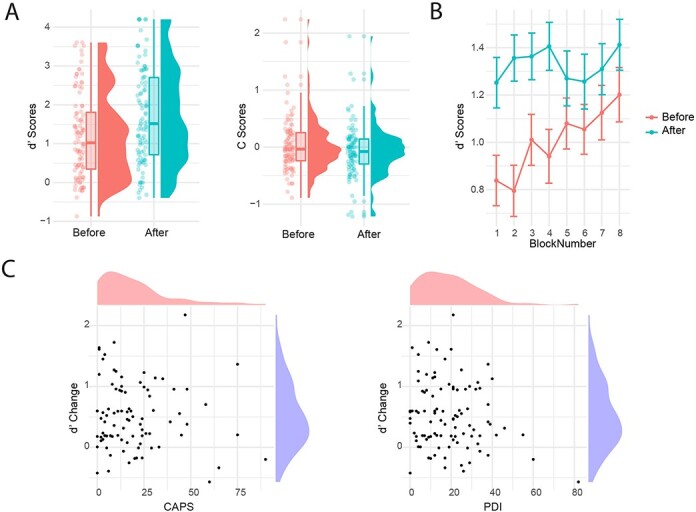
(A) *d′* (left) and C change (right panel) with prior knowledge exposure, (B) *d′* change over time in the common 8 band, +6 dB condition. Note that the grand mean for *d′* values for the by block analyses differs to the main analysis because the adjustment for extreme values was conducted by block in this instance rather than across the whole experiment (Macmillan and Kaplan 1985), (C) showing the lack of evidence in support of a relationship between *d′* change and the CAPS (left) and PDI measures (right panel)

A one-way independent ANOVA with the group as a factor showed that there was no evidence of a difference in d’ between the five data collection groups, so data were pooled for the common 8 bands +6 dB condition (*F* (4, 94) = 1.83, *P* = 0.130, η^2^ = 0.07). Speech identification accuracy in this condition was above chance before prior knowledge exposure (*t*(98) = 10.96, *P* = 9.952e-19), but crucially was significantly increased after prior knowledge exposure (*t*(98) = 9.34, *P* = 3.217e-15, *d* = 0.44, see Fig. 4A and Table 3).

**Table 3.** Signal detection outcomes for the SVS speech detection task for the 8 band, +6 dB condition. Wilcoxon tests were used beta due to non-parametric data

	Before		After				
	*M*	*SD*	*M*	*SD*	*Change statistic T(Z)*	*P*	*D(r)*
d’	1.20	1.09	1.72	1.25	9.34	3.217e-15	0.44
C	0.04	0.48	−0.06	0.45	2.99	0.004	0.22
*β*	1.28	1.23	1.14	0.98	1.59	0.113	0.16

A 2 × 8 repeated measures ANOVA with factors prior knowledge exposure (pre-/post) and block (1–8) was conducted to understand how *d′* changed across the experiment. This indicated a significant exposure × block interaction (*F* (6.07, 595.19) = 2.23, *P* = 0.038, η^2^ = 0.02, see Fig. 4B). Follow-up repeated measure one-way ANOVAs indicated a change in d’ across blocks before exposure to prior knowledge (*F* (6.23, 610.46) = 3.33, *P* = 0.003, η^2^ = 0.03) but not after it (*F* (6.18, 605.54) = 0.86, *P* = 0.530, η^2^ = 0.009). In the pre-exposure phase, the change in accuracy increased linearly with block progression (*F*(1, 98) = 14.83, *p* = 2.100e-4, η^2^ =0.13). There was also the main effect of block such that accuracy in general increased across blocks (*F* (5.94, 582.36) = 2.17, *P* = 0.045, η^2^ = 0.02) and from pre- to post-exposure (*F* (1, 98) = 64.69, *P* = 2.054e-12, η^2^ = 0.40). Hence, even with these more challenging stimuli, participants continued to improve in their ability to detect speech in SVS in the pre-exposure phase, demonstrating the learning opportunity inherent to SWS/SVS.

### Testing for a modulation effect using SVS

As in the previous studies, there was no evidence of a significant relationship between change in d’ and the CAPS (*rs* (88) = −0.02, *P* = 0.852) or the PDI (*rs* (98) = −0.12, *P* = 0.257), see Fig. 4C). One-sided Bayesian correlations assessing a positive association between d’ and the questionnaire measures were used to assess the relative evidence for the null as compared to the experimental hypothesis. These tests provided strong evidence for the null hypothesis (both one-sided BF_01_ > 11). Partial correlation analysis, controlling for baseline discrimination scores, also provided no evidence for an association between d’ change and the CAPS (rs (88) =−0.03, *P* = 0.815) and PDI scores (rs (98) = −0.12, *P* = 0.255). We also tested to see if change in *d′* correlated with either the CAPS or PDI on the additional acoustic conditions (e.g. those differing in number of bands and SNR level), but this was not the case (see Supplementary Materials, Experiment 3), suggesting that varying the difficulty level made no difference to the lack of association with hallucination-proneness.

### Assessing relationships with bias

A decrease in C after prior knowledge exposure reflected an increased bias to report the presence of speech [C (*t* (98) = 2.99, *P* = 0.004, *d* = 0.22)]. However, C did not differ significantly from zero either before or after prior knowledge exposure (both *P*s > 0.194). A non-parametric Wilcoxon test—to account for a deviation in normality—also indicated that beta values did not change significantly (*z *= 1.58, *P* = 0.113). These findings suggest that the SVS stimuli provide a more controlled modulation of discrimination, while holding bias relatively more constant. Beta and C change did not correlate with either the CAPS or PDI frequency measures (all *P*s > 0.369).

Therefore even with a stimulus which is harder to learn and offers a tighter control on learning effects, we found no evidence that explicitly modulating expectations for speech leads to greater gains in discrimination for people who are hallucination prone. Using SVS we were more able to control learning effects—both pre- and post-exposure—and these were still considerable, even with harder stimuli. However, notwithstanding such effects, we were unable to provide evidence for an auditory version of the modulation hypothesis.

## Experiment 3

Replicating and extending the naïve listening effect.

Our difficulties in demonstrating modulation effects in the auditory domain raised the worry that our prior observations with SWS may also be challenging to replicate. It could be that that such stimuli are too unusual or unique in some way, hampering efforts to index top-down effects on perception. The aim of our final experiment therefore was to replicate and extend the ‘naïve listening’ effect observed in our study with NCVH (Alderson-Day *et al.* 2017), but this time using SVS. In that study, participants with frequent experience of hearing voices reported recognising speech in SWS earlier than control participants, despite not being informed that speech was hidden in the stimulus. Those who reported ‘tuning in’ to SWS earlier also reported significantly greater levels of AVH in the preceding week—as measured on the ‘physical characteristics’ subscale of the PSYRATS (Haddock *et al.* 1999)—but they performed no differently to controls on a post-exposure discrimination task, in a similar manner to the other studies reported here.

To reproduce this, we reran the naïve listening procedure and collected hallucination-proneness measures on the LSHS-A (*n* = 134) in a larger, healthy sample of individuals. Moreover, we added an additional procedure to introduce a more objective measure of stimulus decoding. One worry about our original procedure was that it relied on participant self-report, and thus may be open to participants claiming they had heard speech or guessing speech was present without actually decoding it. Here we introduced a memory test that could only be successfully completed if participants had actually been understanding words in the SVS prior to the ‘reveal’ that speech was present. This, therefore, would extend our initial finding by providing more objective evidence of early SVS comprehension. Using this procedure, we predicted that people higher in auditory hallucination-proneness would (i) report recognising speech in the SVS earlier than others, and (ii) be able to remember significantly more words hidden in the SVS task. We also included measures of visual hallucination-proneness and general schizotypal traits—to test for specificity—and a test of SVS discrimination post-exposure (for comparison with our prior study).

## Method

### Participants

We recruited 134 participants (age *M*(*SD*)= 21.45 (5.79), range 18–59 years, 46 male/2 other) via university departments, social media, and word-of-mouth. Exclusion criteria were the same as for the previous experiments. Participants were invited to take part in a ‘study of auditory perception’ that involved listening to some ‘unusual sounds’, but no mention of voices or speech was included in the study materials.

### Materials and procedure

The procedure for experiment 3 closely followed Alderson-Day *et al.* (2017). The measures used are reported here in the order they were attempted by participants.

#### National Adult Reading Test (NART; *Nelson 1982*)

The NART is a measure of vocabulary and reading ability which has been used extensively in research on psychosis as an indicator of premorbid IQ (e.g. Broome *et al.* 2012) and was included in Alderson-Day *et al.* (2017) for group matching. We retained it here to follow that procedure, but also to provide control material for the memory task. By including a small selection of words from the NART (plus similar words matched for unusual spelling), we could control for general memory differences between those who did and did not recognise speech in the SVS.

#### SVS naïve listening procedure

Intelligible and unintelligible SVS stimuli were drawn from the same set as Experiment 2. In Experiment 3, the 16-band, +6 db stimuli were used, as these appeared to be the most similar to SWS in terms of their level of difficulty. Participants were told that they would be listening out for a scratchy target sound that sounded ‘different’ from the other sounds and began the procedure by listening to three examples of the target randomly presented along with five examples of unintelligible SVS.^4^ The target was used to maintain attention and provide an incidental task which would discourage participants guessing at the purpose, i.e. the potential intelligibility of distractors, hidden in the SVS. As in Alderson-Day *et al.* (2017), the ‘scratchy’ sounds were examples of unintelligible SWS (i.e. the frequency and amplitude tracks of two separate original sentences combined), that had been further noise-vocoded, giving them a different timbre and sound quality. Once participants could discriminate the two kinds of sound, they attempted the main listening task, which contained six blocks of 15 SVS stimuli (45 intelligible, 45 unintelligible) and three targets per block. At the start of each block, a visual stimulus appeared announcing the start of the block (i.e. block 1, block 2, etc). Stimuli were presented in a predefined pseudo-random order with no more than two of the same kind of stimuli consecutively.

Once the participants had listened to all six blocks, they were asked by the experimenter (i) if they noticed anything unusual about the words, (ii) if they noticed any words and sentences and crucially (iii) if they knew which round they started noticing them (using the visual block markers as means of marking out time). Participants’ estimates for the third question were used as the main task outcome, defined as their ‘recognition point’. They were then told that in fact there were words present in the stimuli, and asked to complete the memory task for words contained in the SVS. The memory test consisted of 46 words, including 18 words included in the SVS (3 per block), 18 words matched for length and complexity that did not feature in the SVS, five words from the NART, and five non-target words matched to the NART words for their irregular spelling. Following recognition memory methodology (Tulving 1985), participants were asked to indicate for each word whether they explicitly *remembered* the word (R), felt like they *knew* they had heard the word at some point (K), or new items that they didn’t recognize (N). As we were most interested in participants actually being able to decode the words, we focused on *remember* scores. New items, in contrast, acted as lures for potential false positives.

As in Alderson-Day *et al.* (2017), participants also then received training in understanding SVS, and then attempted an accuracy task (testing their speech/non-speech discrimination and accuracy for understanding keywords in 25 intelligible and 25 unintelligible SVS trials). Results for these tasks are included in Supplementary Materials.

#### Questionnaires

Following the tasks, participants completed the LSHS-A (as in Experiment 1b), but also two further measures: the four corresponding visual questions from the LSHS (i.e. the LSHS-V) and the brief version of the Oxford Liverpool Inventory of Feelings and Experiences (OLIFE; Mason and Claridge 2006; Fernyhough *et al.* 2008). This allowed for specificity testing by comparing auditory hallucination-proneness with visual experiences and general proneness to schizotypal experiences.

## Results and discussion

### Assessing naïve listening via self-report

No participants guessed the purpose of the experiment before testing. Overall, 83/134 participants (62%) recognized speech being present in the SVS and 51 (38%) did not. Participants that recognized speech did so most frequently in the second block (mean = 2.58, median = 3, range = 1–5) and of those, 72 were able to repeat some of the words encoded in the stimuli.

We wanted to assess how well the LSHS-A predicted which participants recognised the speech without training. In a logistic regression model, LSHS-A scores significantly predicted recognition group (*Z* = 2.206, *P* = 0.027, OR = 1.21, CI = 1.03–1.44). Specificity analysis swapping LSHS-A for visual items of the Launay–Slade (LSHS-V) or a measure of general schizotypy (the OLIFE) did not result in significant models (see Supplementary Materials).

The ability to decode ambiguous speech stimuli and similar skills (such as extracting speech from noise) are known to vary considerably across individuals. To explore the naïve listening effect, we ran an exploratory analysis drawing on the NART scores collected at the start of the experiment. Along with offering a very rough proxy of verbal intelligence, NART scores reflect vocabulary and reading ability—all of which would plausibly contribute to the identification of ambiguous speech. We therefore reran our logistic regression analyses, but including NART as an additional predictor. Higher NART scores were associated with identifying speech spontaneously (*Z* = 3.78, *P* < 0.001, OR = 1.15, CI = 1.08–1.25) but the contribution of LSHS-A scores was now non-significant (*Z* = 1.80, *P* = 0.072, OR = 1.17, CI = 0.99–1.40), despite similar odds ratios for the two predictors.

### Assessing naïve listening via memory performance

Following the reveal that speech was present participants were tested on their memory for hidden words, to provide a more objective test of spontaneous SVS decoding. Across the whole sample (i.e. combining those who did and did not recognize speech spontaneously), accuracy for ‘remember’ items on the memory task significantly correlated with LSHS-A scores, *rs* = 0.20, *P* = 0.020 (Spearman’s test).

This relation could feasibly have been driven by a general tendency to endorse words on the memory test (i.e. a false positive bias), or general differences in memory scores. If so, LSHS-A scores would have also correlated with (i) endorsement of new (i.e. lure) items on the memory task, or (ii) recall for NART items included in the memory task. However, neither new item scores, *r* = -0.00, *P* = 0.969, nor for memory for NART words, *r* = 0.05, *P* = 0.551, were associated with LSHS-A scores. This suggested that the ability to understand the SVS early, and thus encode more hidden words, was indeed related to greater hallucination-proneness.

Given the apparent role of NART scores in predicting self-reported recognition of SVS, we used partial correlation tests to assess what role it played in the relationship between LSHS-A scores and ‘remember’ scores for words hidden in the SVS. LSHS-A scores positively correlated with words remembered when controlling for the NART, *r* = 0.18, *P* = 0.035), but no significant relation between memory performance and NART scores was evident when controlling for LSHS-A, *r* = 0.13, *P* = 0.136.

The results of Experiment 4 therefore supported the original findings of Alderson-Day *et al.* (2017), but added to it by providing a more objective test of participants being able to recognize speech early—namely, actual improved recall for words hidden in the SVS. Moreover, this was specific to auditory hallucination-proneness and not visual hallucination-proneness, or general schizotypy.

## General discussion

Our aim in the present paper was to align and reconcile two findings of enhanced top-down perceptual processing in people prone to hallucinations. Across four experiments, we were only partially successful in this aim. In Experiments 1a, 1b and 2 we could not provide evidence for a modulation hypothesis in the auditory domain. People who were more prone to hallucinations did not appear to draw upon prior expectation more in their perception when their expectations were explicitly updated. In contrast, in Experiment 3 we provided further evidence for a naïve listening effect: healthy people who were prone to hallucinations were more able than others to identify speech in SVS without knowing that speech was present.

This pattern of results suggests a spontaneous, rather than directed, use of top-down resources in people who are prone to unusual perceptions, in contrast to prior findings using visual paradigms (Teufel *et al.* 2015). How to explain this discrepancy? First, it is important to consider the potential differences in the kind of information provided by each stimulus. As already noted, SWS/SVS appeared to allow for learning across trials, even in a naïve state. This represents an opportunity for perceptual learning (Sohoglu and Davis 2016) that would not appear to be apparent when learning to discriminate Mooney images. Moreover, it raises the possibility that some participants may learn to discriminate speech from non-speech without understanding the content of the speech (doing so possibly via prosodic or structural cues). As such, discriminating speech from non-speech in SWS/SVS may be posing a different kind of challenge to Mooney images, and may not be solely reliant on top-down knowledge.

This might raise the concern that SWS/SVS stimuli are just *too* different from degraded images to explore top-down effects in a comparable way. However, when discrimination was made especially challenging and learning effects minimized (in experiment 2), we still observed no evident relationship between hallucination-proneness and template exposure. Moreover, in our final experiment, people who were more AVH-prone were specifically more able to remember hidden words in the SVS, suggesting they could successfully decode the stimuli (rather than discriminating speech from non-speech in a more general way). If the difference between auditory and visual stimuli of this kind is simply one of difficulty, or the number of generalizable cues across trials, then this can be explored empirically in future studies. It would be possible to parametrically vary the perceptibility of hidden speech in SVS by adapting the signal-to-noise ratio and reducing the number of vocoding channels. This would dampen such effects and could demonstrate modulatory effects that would be comparable to those seen in the visual domain.

If the lack of any modulation effect is genuine, however, it might suggest that healthy people prone to unusual experiences are not necessarily susceptible to momentary effects—such as suggestibility, or demand characteristics—and instead possess perceptual biases that are somehow more ingrained. In predictive processing terms, this could constitute a higher-order belief about the world (e.g. ‘the universe is full of hidden meanings’) which directs the individual response in ambiguous situations to explore potentially important signals despite the instruction to listen for the target sound. Exploring differences in attention during naïve listening will be vital, as some participants attitude towards unusual and ambiguous stimuli may lead them to allocate more attention to SVS, thus giving them a greater opportunity to engage in explicit or implicit processes of perceptual learning. Recent research on primal world beliefs represents a promising avenue for this line of research (Clifton *et al.* 2019).

Alternatively, hallucinations may relate to a very different level in the processing hierarchy and be expressed in how auditory objects are represented. A recent reframing of the predictive approach by Teufel and Fletcher (2020) has proposed to separate long-term, context invariant ‘constraints’ on perception from context-specific, temporary ‘expectations’ that shape immediate, moment-to-moment sensation. Our data point towards a long-term constraint for some individuals in how they recognise auditory objects when faced with ambiguous speech-like stimuli, rather than sensitivity to changes in situation-specific expectations. Support for this argument comes from recent work on the templates that people use to make judgements about speech. By creating ‘speechiness’ kernels for individual participants, Erb and colleagues (2020) have demonstrated that people high in hallucination-proneness utilize qualitatively different speech templates when discriminating speech sounds, such that lower frequencies typical of speech are attended to less, compared to higher frequency auditory information. If so, this suggests a fundamental long-term alteration to how speech is recognised and processed in those prone to hallucinatory and illusory experiences—rather than a dynamic, moment-to-moment volatility in how expectations are managed. Variation of naïve listening effects with SVS tailored to different speech kernels could be used to explore this further.

Finally, such effects are likely to be shaped by a considerable number of individual differences, including auditory experience and cognitive factors such as verbal IQ (Gwilliams and Wallisch 2020). We explored the role of NART scores in our final experiment for this reason. The observed relationship between those scores and spontaneous recognition suggests it may have a significant impact on SVS discrimination, with implications for future studies. Importantly, the same relationship was not evident for performance on the memory task—suggesting that verbal IQ differences could not fully account for the relationship between SVS decoding and hallucination-proneness. Nevertheless, the range of variables guiding individual comprehension skills for degraded speech, noise-vocoded speech and speech-in-noise are vast, complex and yet to be clearly pinpointed (McGettigan *et al.* 2012).

Some limitations of our general approach must be noted. First, we tested exclusively university and general population-based samples, rather than either clinical or NCVH (i.e. those with very regular hallucination-like experiences; Johns *et al.* 2014). We therefore cannot rule out that participants with more frequent experiences would not show specific modulation effects on their perception. However, predictive approaches to hallucinations are often explicitly framed as models of both clinical and non-clinical phenomena, with the former resulting from an accentuation of mechanisms underlying normal, veridical perception rather than being specific to clinical disorders (Corlett *et al.* 2019). Our data therefore would appear relevant to understanding perceptual mechanisms across the psychosis continuum.

Second, none of our experiments deployed basic tests of auditory processing or hearing ability, which could contribute to low-level differences in how SWS/SVS are recognized and processed. There is emerging evidence that subtle differences in hearing ability can be associated with hallucinations for some people (Linszen *et al.* 2016). Participants with a self-declared hearing difficulty were not recruited to the study, and tests of speech intelligibility do not pose the same challenges to hearing as other tasks used in hallucination research (such as auditory signal detection), but closer control of these factors would have been to test hearing skills in all participants.

An open question for future research is how people with frequent and distressing hallucinations perform under naïve listening conditions and how a perceptual advantage in some conditions translates into non-veridical experiences in other contexts. A recent small-scale study by Kafadar and colleagues (2020)—using the structure of Experiment 1a but the same stimuli as Experiments 2 and 3—found that people at clinical high risk of psychosis are marked more by their pre-exposure *bias* to hear speech, rather than discrimination. They also found no differences in post-exposure discrimination, suggesting again that the explicit modulation of expectation was less relevant to understanding how they perceived SVS. It may be that healthy individuals who have unusual experiences have very slight biases in their perception that facilitate a top-down advantage, akin to the concept of encoding style for internal over external meanings^5^ (Valérie *et al.* 2011). Conversely, individuals with clinical hallucinations may be more fixed in their expectations of finding signal in noise, leading to non-veridical experiences more generally. In such a situation, a slight bias might yield an advantage—especially if it can be applied selectively—whereas a strong bias would lead to false positives (i.e. hearing speech in unintelligible SWS/SVS).

In conclusion, the experiments that we present here refine our understanding of how top-down expectations shape speech perception for people prone to auditory hallucinations. Directly updating expectation would not appear to confer an advantage for people higher in hallucination-proneness—at least for these kinds of stimuli—but being susceptible to hallucinations would appear to be associated with responding to ambiguous speech stimuli in a differential way, facilitating speech identification. This implicates longer term and potentially lower-level constraints on how speech is recognized in such populations, rather than a temporary sensitivity to expectation.

## Supplementary Material

niac002_Supp

## Footnotes

1 While a convenience sample, this number was nevertheless sufficiently powered to identify an effect in a similar range to Teufel and colleagues’ observed effect size for the CAPS (*r* = 0.42, 90% power, min. sample = 50).

2 Rosen and colleagues’ SWS stimuli were also noise-vocoded. This step was omitted in our use of the stimuli for the present paper and in Alderson-Day *et al.* (2017), as noise-vocoding can induce an effect akin to whispering, and can make the underlying sinewaves cohere—both of which could potentially reveal the underlying speech signal.

3 A Bayesian mixed ANOVA in JASP indicated strong evidence for an exposure effect (BF = 5.90) and weak evidence for either an effect of priming group (BF = 0.44) or an exposure*prime interaction (BF = 0.43; BF inclusion statistics reported).

4 Specifically, participants were told: *‘Your task is to listen out for a target sound and press the space bar every time you hear it. It might sound “scratchy” compared to the others. Have a listen to these examples, and press the space bar if you think you hear the unusual sound.’*

5 Kristiina Kompus (U. Bergen) is thanked for this comparison.
