# Supplementary material for: Susceptibility to auditory hallucinations is associated with spontaneous but not directed modulation of top-down expectations for speech
Source: Neurosci Conscious. 2022 Feb 1;2022(1):niac002. doi: 10.1093/nc/niac002 (PMC8824703; doi:10.1093/nc/niac002)
Supplement: niac002_Supp [file niac002_supp.zip › Supplementary materials v3 - Alderson-Day Evans 2022.pdf]

Supplementary materials for Alderson-Day et al. (2021) "Susceptibility to auditory hallucinations is associated with spontaneous but not directed modulation of top-down expectations for speech"

Experiment 1a

Table 1: Correlations between questionnaire variables and task performance on SWS discrimination pre- and post-exposure.

			**Pre-exposure**			**Post-exposure**		
		PDI	β	C	d'	β	C	d'
	CAPS	0.77***	-0.21	-0.08	-0.17	-0.04	-0.12	-0.14
	PDI	1	-0.04	0.02	-0.13	-0.09	-0.18	-0.03
**Pre**	β		1	0.67***	0.13	0.12	0.13	-0.03
	C			1	-0.29*	0.38**	0.31*	-0.19
	d'				1	-0.38**	-0.33**	0.68***
**Post**	β					1	0.9***	-0.38**
	C						1	-0.34**
	d'							1

CAPS = Cardiff Anomalous Perception Scale, PDI = Peters Delusion Inventory.
* p <0.05 ** p <0.01 *** p <0.001

Experiment 1b

Note 1. Bias measures following listening prime task

No differences were observed between the primed (M(SD) = 0.38 (0.53)) and control groups (M(SD) = 0.20 (0.72)) on pre-exposure C (t (53.67) = -1.0359, p = 0.3049). Similarly, no group differences were observed for pre-exposure beta scores (Primed M(SD) = 2.68 (2.83); Control M(SD) = 2.48 (3.28); t(56.80)= -0.258, p = 0.797).

Experiment 2

Figure 1: Further analyses of the additional auditory conditions

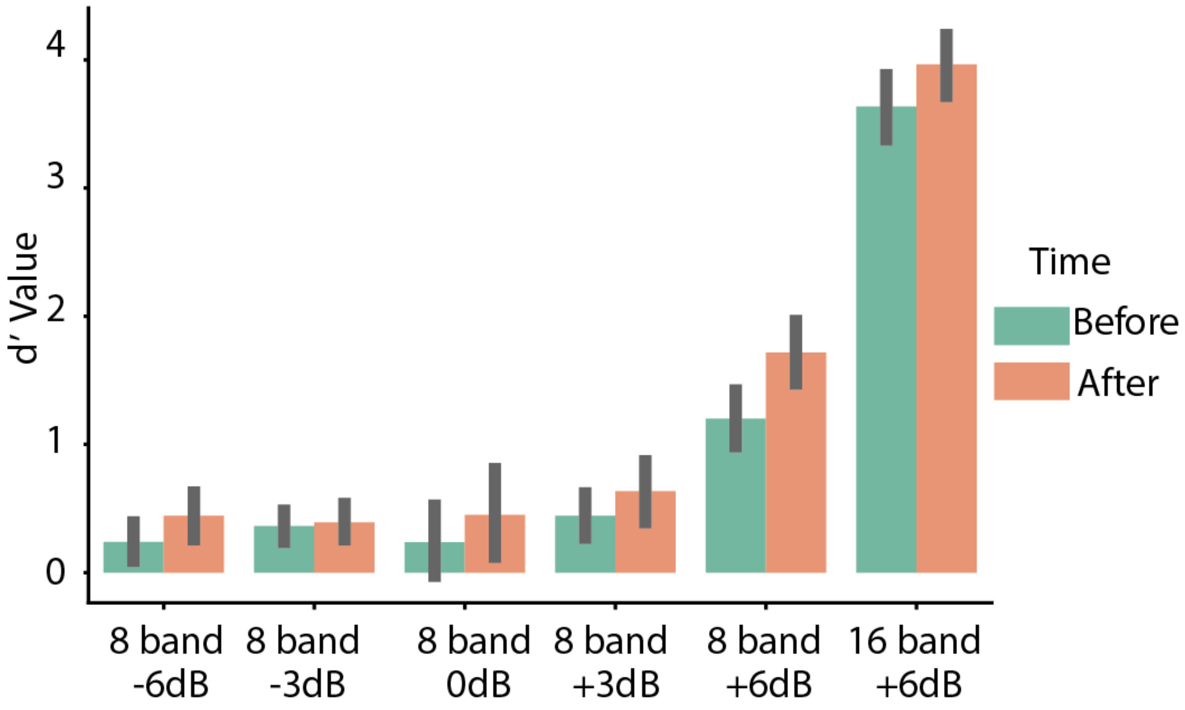

The association between d' values and the questionnaire measures were also examined in the additional acoustic conditions shown above. Speech identification accuracy depended on the SNR and the number of bands. Note that these samples were relatively small, containing around 20 participants each. There was no significant correlation between d' and CAPS (all *ps* > 0.382) or the PDI (all *ps* > 0.126) in any condition.

Experiment 3

Note 2. Specificity testing for visual hallucination-proneness (LSHS-V) and general schizotypy (OLIFE)

A model was tested containing age, gender, and Launay-Slade Visual Hallucination (LSHS-V) scores as predictors of recognition group. The resulting model was non-significant (X(1) = 1.33, p = 0.10), with LSHS-V making no significant contribution to the model (Z = 1.58, p = .115, OR = 1.18, CI = 0.97-1.446. When scores for general schizotypy were included instead, a very similar model was produced: a non-significant overall fit (X(1) = 1.33, p = 0.249 and

no association between recognition group and OLIFE (Z = 1.15, p = .252, OR = 1.04, CI = 0.97-1.13).

Note 3. Comparing participants who did and did not recognised speech spontaneously on subsequent task performance.

To allow for comparison with prior applications of SWS/SVS, we trained participants after the naïve listening task to understand SVS and tested their accuracy. Training consisted of listening to six new sentences and receiving visual feedback as their underlying meaning. Following Alderson-Day, Lima et al. (2017), participants were then tested for their speech/non-speech discrimination and keyword comprehension for the 50 SVS trials (25 intelligible, 25 unintelligible), 45 of which appeared in the initial listening task. Signal detection outcomes were again calculated to assess d' and beta scores, post-training.

As with the memory task, these others measures indicated that people who recognised SVS spontaneously showed further benefits in SVS training and discrimination. Table 2 below shows that "recognisers" also reported "knowing" more words on the memory test (a less explicit test of their memory for SVS words), showed greater ability to understand SVS during training, and were more able to discriminate speech from non-speech on the final accuracy task (including understanding more keywords). This would support the argument that their self-reported ability to decode the speech was genuine and facilitated subsequent perceptual performance. However, recognisers also showed a greater bias towards stating that speech was present on the accuracy task, as indicated by smaller beta and C values.

Table 2. Memory and accuracy task performance compared by recognition group.

	Recognised speech (n = 83)		Did not recognise (n = 51)				
	M	*SD*	*M*	*SD*	*T(Z)*	*p*	*d(r)*
Memory test							
Recognised	5.43	3.16	3.76	1.49	4.13	6.65e-05	0.73
Knew	4.82	3.95	2.90	3.59	2.89	.005	0.51
New	16.19	5.10	16.94	5.93	-0.75	.457	-0.13
NART words	4.16	1.42	4.41	1.02	-1.21	.230	-0.22
Accuracy task							
D'	2.72	0.89	1.60	1.22	5.73	1.596e-07	1.02
C	-0.21	0.52	0.02	0.49	-2.62	.010	-0.47

Beta*	1.23	1.64	1.41	1.29	2.75	.006	0.24
Keywords	39.65	17.03	15.04	14.70	8.85	1.036e-14	1.57

*Wilcoxon test used due to non-normal data. Brackets indicate equivalent non-parametric tests used.
